# Supplementary material for: Estimands and their implications for evidence synthesis for oncology: A simulation study of treatment switching in meta-analysis
Source: Res Synth Methods. 2025 Oct 16;17(1):170–93. doi: 10.1017/rsm.2025.10039 (PMC12824772; doi:10.1017/rsm.2025.10039)
Supplement: Metcalfe et al. supplementary material [file S1759287925100392sup001.docx]

Appendix of Estimands and Their Implications for Evidence Synthesis for Oncology: A Simulation Study of Treatment Switching in Meta-Analysis

# Overview

In Appendix [Section 1](#sec-censor), the details of censoring switchers as an analytical strategy to estimate the hypothetical estimand are provided.

In Appendix [Section 2](#sec-sensMain), the results of the sensitivity analyses of the main simulation setting are presented. In particular, Appendix [Section 2.1](#sec-sensMain2) presents the results where the hypothetical estimand is estimated using a Rank-Preserving Structural Failure Time Model (RPSFTM), under a fixed-effects data-generating mechanism and fixed-effects meta-analytical estimation. Appendix [Section 2.2](#sec-sensMain3) presents the results where the hypothetical estimand is estimated using RPSFTM, under a random-effects data-generating mechanism and random-effects meta-analytical estimation.

In Appendix [Section 3](#sec-supp), the results of the supplementary simulations where switchers were censored at the time of switch to estimate the hypothetical estimand are presented. Appendix Section 3.1 presents the results under a fixed-effects data-generating mechanism and random-effects meta-analytical estimation. Appendix Section 3.2 presents results under a fixed-effects data-generating mechanism and fixed-effects meta-analytical estimation; and Appendix Section 3.3 presents results under a random-effects data-generating mechanism and random-effects meta-analytical estimation.

# 1. Details of censoring switchers

In the supplementary simulations, censoring switchers was used as the analytical strategy to estimate the hypothetical estimand at the trial level. In this strategy, a Cox proportional hazards model with OS as the outcome and treatment as a covariate was fit to the OS times of patients in the control and treatment groups, but the OS times of switchers in the control group were censored at the time of switching. We extracted the estimated log HR and standard error using the same approach as in the estimation of the treatment policy estimand.

# 2. Sensitivity analyses of main simulation

## 2.1 Fixed-effects data-generating mechanism, fixed-effects meta-analysis

In this section, we present the results of the sensitivity analysis of the main simulation setting where estimates were pooled using a fixed-effects meta-analysis. A fixed-effects model was assumed when generating the trial data.

| Table 1: Averages of pooled treatment effect estimates and comparison against treatment policy estimand under an assumed HR of 0.60 for the transition hazards of the illness-death model in the simulation with fixed-effects data-generating mechanism, fixed-effects meta-analysis, and rank-preserving structural failure time models   \| **Scenarios** \| **Estimators** \| **Estimated treatment effects:  HR (averaged 95% CI)** \| **Bias  (2.5%, 97.5% difference percentiles)** \| **Coverage** \| \| --- \| --- \| --- \| --- \| --- \| \| **75% switching rate for control arm** \|  \|  \| **Comparison against treatment policy estimand (True HR = 0.66)** \| \| \| 2:1 allocation,  75% switching rate for control arm^a^ \| Pure HE (100%) \| 0.41 (0.34, 0.51) \| -0.25 (-0.30, -0.17) \| 0.00 \| \| Mixed TPE (25%) and HE (75%) \| 0.55 (0.48, 0.64) \| -0.11 (-0.18, -0.02) \| 0.31 \| \| Mixed TPE (50%) and HE (50%) \| 0.61 (0.54, 0.69) \| -0.05 (-0.12, 0.02) \| 0.72 \| \| Mixed TPE (75%) and HE (25%) \| 0.64 (0.58, 0.71) \| -0.02 (-0.09, 0.05) \| 0.90 \| \| Pure TPE (100%) \| 0.66 (0.60, 0.72) \| -0.00 (-0.06, 0.06) \| 0.95 \| \| 1:1 allocation,  75% switching rate for control arm \| Pure HE (100%) \| 0.41 (0.33, 0.50) \| -0.25 (-0.31, -0.18) \| 0.00 \| \| Mixed TPE (25%) and HE (75%) \| 0.55 (0.48, 0.64) \| -0.11 (-0.18, -0.03) \| 0.29 \| \| Mixed TPE (50%) and HE (50%) \| 0.61 (0.54, 0.69) \| -0.05 (-0.12, 0.03) \| 0.73 \| \| Mixed TPE (75%) and HE (25%) \| 0.64 (0.58, 0.71) \| -0.02 (-0.08, 0.05) \| 0.92 \| \| Pure TPE (100%) \| 0.66 (0.61, 0.72) \| 0.00 (-0.05, 0.06) \| 0.95 \| \| **50% switching rate for control arm** \|  \|  \| **Comparison against treatment policy estimand (True HR = 0.64)** \| \| \| 2:1 allocation,  50% switching rate for control arm \| Pure HE (100%) \| 0.53 (0.46, 0.60) \| -0.11 (-0.17, -0.05) \| 0.15 \| \| Mixed TPE (25%) and HE (75%) \| 0.57 (0.50, 0.64) \| -0.07 (-0.13, -0.00) \| 0.49 \| \| Mixed TPE (50%) and HE (50%) \| 0.60 (0.54, 0.67) \| -0.04 (-0.10, 0.02) \| 0.76 \| \| Mixed TPE (75%) and HE (25%) \| 0.62 (0.56, 0.68) \| -0.02 (-0.08, 0.04) \| 0.90 \| \| Pure TPE (100%) \| 0.64 (0.58, 0.70) \| -0.00 (-0.06, 0.06) \| 0.95 \| \| 1:1 allocation,  50% switching rate for control arm \| Pure HE (100%) \| 0.52 (0.46, 0.59) \| -0.12 (-0.18, -0.05) \| 0.11 \| \| Mixed TPE (25%) and HE (75%) \| 0.57 (0.51, 0.64) \| -0.07 (-0.13, -0.01) \| 0.45 \| \| Mixed TPE (50%) and HE (50%) \| 0.60 (0.54, 0.66) \| -0.04 (-0.10, 0.02) \| 0.77 \| \| Mixed TPE (75%) and HE (25%) \| 0.62 (0.57, 0.68) \| -0.02 (-0.07, 0.04) \| 0.91 \| \| Pure TPE (100%) \| 0.64 (0.59, 0.70) \| 0.00 (-0.06, 0.06) \| 0.95 \| \| **^a^This table shows estimated treatment effects under an assumed hazard ratio (HR) of 0.60 for the transition hazards of the illness-death model and bias and coverage in comparison to the true treatment policy estimand. Monte Carlo standard errors for all measures are very close to zero  Acronyms: CI: Confidence intervals; HE - Hypothetical estimator; HR - Hazard ratio; TPE - Treatment policy estimator.** \| \| \| \| \| |
| --- | --- | --- | --- | --- | --- | --- | --- | --- | --- | --- | --- | --- | --- | --- | --- | --- | --- | --- | --- | --- | --- | --- | --- | --- | --- | --- | --- | --- | --- | --- | --- | --- | --- | --- | --- | --- | --- | --- | --- | --- | --- | --- | --- | --- | --- | --- | --- | --- | --- | --- | --- | --- | --- | --- | --- | --- | --- | --- | --- | --- | --- | --- | --- | --- | --- | --- | --- | --- | --- | --- | --- | --- | --- | --- | --- | --- | --- | --- | --- | --- | --- | --- | --- | --- | --- | --- | --- | --- | --- | --- | --- | --- | --- | --- | --- | --- | --- | --- | --- | --- | --- | --- | --- | --- |

| Table 2: Averages of pooled treatment effect estimates and comparison against treatment policy estimand under an assumed HR of 0.80 for the transition hazards of the illness-death model in the simulation with fixed-effects data-generating mechanism, fixed-effects meta-analysis, and rank-preserving structural failure time models   \| **Scenarios** \| **Estimators** \| **Estimated treatment effects:  HR (averaged 95% CI)** \| **Bias  (2.5%, 97.5% difference percentiles)** \| **Coverage** \| \| --- \| --- \| --- \| --- \| --- \| \| **75% switching rate for control arm** \|  \|  \| **Comparison against treatment policy estimand (True HR = 0.84)** \| \| \| 2:1 allocation,  75% switching rate for control arm^a^ \| Pure HE (100%) \| 0.66 (0.53, 0.82) \| -0.18 (-0.32, 0.00) \| 0.36 \| \| Mixed TPE (25%) and HE (75%) \| 0.77 (0.66, 0.89) \| -0.07 (-0.19, 0.06) \| 0.75 \| \| Mixed TPE (50%) and HE (50%) \| 0.81 (0.71, 0.91) \| -0.03 (-0.13, 0.07) \| 0.87 \| \| Mixed TPE (75%) and HE (25%) \| 0.83 (0.74, 0.92) \| -0.01 (-0.10, 0.08) \| 0.92 \| \| Pure TPE (100%) \| 0.84 (0.76, 0.92) \| -0.00 (-0.08, 0.08) \| 0.95 \| \| 1:1 allocation,  75% switching rate for control arm \| Pure HE (100%) \| 0.66 (0.53, 0.81) \| -0.18 (-0.31, -0.00) \| 0.34 \| \| Mixed TPE (25%) and HE (75%) \| 0.77 (0.67, 0.89) \| -0.07 (-0.18, 0.06) \| 0.75 \| \| Mixed TPE (50%) and HE (50%) \| 0.81 (0.72, 0.91) \| -0.03 (-0.12, 0.07) \| 0.89 \| \| Mixed TPE (75%) and HE (25%) \| 0.83 (0.75, 0.92) \| -0.01 (-0.09, 0.08) \| 0.93 \| \| Pure TPE (100%) \| 0.84 (0.77, 0.92) \| 0.00 (-0.07, 0.08) \| 0.95 \| \| **50% switching rate for control arm** \|  \|  \| **Comparison against treatment policy estimand (True HR = 0.83)** \| \| \| 2:1 allocation,  50% switching rate for control arm \| Pure HE (100%) \| 0.75 (0.65, 0.86) \| -0.08 (-0.18, 0.07) \| 0.63 \| \| Mixed TPE (25%) and HE (75%) \| 0.78 (0.69, 0.88) \| -0.05 (-0.14, 0.07) \| 0.79 \| \| Mixed TPE (50%) and HE (50%) \| 0.80 (0.72, 0.89) \| -0.03 (-0.11, 0.08) \| 0.87 \| \| Mixed TPE (75%) and HE (25%) \| 0.81 (0.74, 0.90) \| -0.01 (-0.09, 0.08) \| 0.92 \| \| Pure TPE (100%) \| 0.82 (0.75, 0.91) \| -0.00 (-0.08, 0.08) \| 0.95 \| \| 1:1 allocation,  50% switching rate for control arm \| Pure HE (100%) \| 0.75 (0.65, 0.86) \| -0.08 (-0.18, 0.05) \| 0.63 \| \| Mixed TPE (25%) and HE (75%) \| 0.78 (0.69, 0.88) \| -0.04 (-0.14, 0.06) \| 0.80 \| \| Mixed TPE (50%) and HE (50%) \| 0.80 (0.72, 0.89) \| -0.02 (-0.11, 0.07) \| 0.89 \| \| Mixed TPE (75%) and HE (25%) \| 0.82 (0.74, 0.90) \| -0.01 (-0.09, 0.07) \| 0.93 \| \| Pure TPE (100%) \| 0.83 (0.76, 0.90) \| 0.00 (-0.07, 0.08) \| 0.95 \| \| **^a^This table shows estimated treatment effects under an assumed hazard ratio (HR) of 0.80 for the transition hazards of the illness-death model and bias and coverage in comparison to the true treatment policy estimand. Monte Carlo standard errors for all measures are very close to zero  Acronyms: CI: Confidence intervals; HE - Hypothetical estimator; HR - Hazard ratio; TPE - Treatment policy estimator.** \| \| \| \| \| |
| --- | --- | --- | --- | --- | --- | --- | --- | --- | --- | --- | --- | --- | --- | --- | --- | --- | --- | --- | --- | --- | --- | --- | --- | --- | --- | --- | --- | --- | --- | --- | --- | --- | --- | --- | --- | --- | --- | --- | --- | --- | --- | --- | --- | --- | --- | --- | --- | --- | --- | --- | --- | --- | --- | --- | --- | --- | --- | --- | --- | --- | --- | --- | --- | --- | --- | --- | --- | --- | --- | --- | --- | --- | --- | --- | --- | --- | --- | --- | --- | --- | --- | --- | --- | --- | --- | --- | --- | --- | --- | --- | --- | --- | --- | --- | --- | --- | --- | --- | --- | --- | --- | --- | --- | --- |

| Table 3: Averages of pooled treatment effect estimates and comparison against treatment policy estimand under an assumed HR of 1.00 for the transition hazards of the illness-death model in the simulation with fixed-effects data-generating mechanism, fixed-effects meta-analysis, and rank-preserving structural failure time models   \| **Scenarios** \| **Estimators** \| **Estimated treatment effects:  HR (averaged 95% CI)** \| **Bias  (2.5%, 97.5% difference percentiles)** \| **Coverage** \| \| --- \| --- \| --- \| --- \| --- \| \| **75% switching rate for control arm** \|  \|  \| **Comparison against treatment policy estimand (True HR = 1.00)** \| \| \| 2:1 allocation,  75% switching rate for control arm^a^ \| Pure HE (100%) \| 0.99 (0.79, 1.24) \| -0.01 (-0.23, 0.25) \| 0.88 \| \| Mixed TPE (25%) and HE (75%) \| 0.99 (0.85, 1.16) \| -0.01 (-0.15, 0.16) \| 0.90 \| \| Mixed TPE (50%) and HE (50%) \| 1.00 (0.88, 1.13) \| -0.00 (-0.12, 0.13) \| 0.92 \| \| Mixed TPE (75%) and HE (25%) \| 1.00 (0.90, 1.11) \| -0.00 (-0.11, 0.11) \| 0.93 \| \| Pure TPE (100%) \| 1.00 (0.91, 1.10) \| -0.00 (-0.10, 0.10) \| 0.95 \| \| 1:1 allocation,  75% switching rate for control arm \| Pure HE (100%) \| 1.00 (0.80, 1.24) \| -0.00 (-0.21, 0.25) \| 0.89 \| \| Mixed TPE (25%) and HE (75%) \| 1.00 (0.86, 1.16) \| -0.00 (-0.14, 0.16) \| 0.90 \| \| Mixed TPE (50%) and HE (50%) \| 1.00 (0.89, 1.13) \| 0.00 (-0.11, 0.13) \| 0.92 \| \| Mixed TPE (75%) and HE (25%) \| 1.00 (0.90, 1.11) \| 0.00 (-0.10, 0.11) \| 0.93 \| \| Pure TPE (100%) \| 1.00 (0.91, 1.10) \| 0.00 (-0.09, 0.10) \| 0.95 \| \| **50% switching rate for control arm** \|  \|  \| **Comparison against treatment policy estimand (True HR = 1.00)** \| \| \| 2:1 allocation,  50% switching rate for control arm \| Pure HE (100%) \| 0.99 (0.86, 1.15) \| -0.01 (-0.15, 0.16) \| 0.85 \| \| Mixed TPE (25%) and HE (75%) \| 1.00 (0.88, 1.13) \| -0.00 (-0.13, 0.14) \| 0.88 \| \| Mixed TPE (50%) and HE (50%) \| 1.00 (0.89, 1.12) \| -0.00 (-0.11, 0.12) \| 0.90 \| \| Mixed TPE (75%) and HE (25%) \| 1.00 (0.90, 1.11) \| -0.00 (-0.10, 0.11) \| 0.92 \| \| Pure TPE (100%) \| 1.00 (0.91, 1.10) \| -0.00 (-0.09, 0.10) \| 0.95 \| \| 1:1 allocation,  50% switching rate for control arm \| Pure HE (100%) \| 1.00 (0.87, 1.15) \| -0.00 (-0.14, 0.16) \| 0.86 \| \| Mixed TPE (25%) and HE (75%) \| 1.00 (0.89, 1.13) \| 0.00 (-0.12, 0.13) \| 0.88 \| \| Mixed TPE (50%) and HE (50%) \| 1.00 (0.90, 1.11) \| 0.00 (-0.10, 0.11) \| 0.91 \| \| Mixed TPE (75%) and HE (25%) \| 1.00 (0.91, 1.10) \| 0.00 (-0.09, 0.10) \| 0.93 \| \| Pure TPE (100%) \| 1.00 (0.91, 1.10) \| 0.00 (-0.09, 0.09) \| 0.95 \| \| **^a^This table shows estimated treatment effects under an assumed hazard ratio (HR) of 1.00 for the transition hazards of the illness-death model and bias and coverage in comparison to the true treatment policy estimand. Monte Carlo standard errors for all measures are very close to zero  Acronyms: CI: Confidence intervals; HE - Hypothetical estimantor; HR - Hazard ratio; TPE - Treatment policy estimantor.** \| \| \| \| \| |
| --- | --- | --- | --- | --- | --- | --- | --- | --- | --- | --- | --- | --- | --- | --- | --- | --- | --- | --- | --- | --- | --- | --- | --- | --- | --- | --- | --- | --- | --- | --- | --- | --- | --- | --- | --- | --- | --- | --- | --- | --- | --- | --- | --- | --- | --- | --- | --- | --- | --- | --- | --- | --- | --- | --- | --- | --- | --- | --- | --- | --- | --- | --- | --- | --- | --- | --- | --- | --- | --- | --- | --- | --- | --- | --- | --- | --- | --- | --- | --- | --- | --- | --- | --- | --- | --- | --- | --- | --- | --- | --- | --- | --- | --- | --- | --- | --- | --- | --- | --- | --- | --- | --- | --- | --- |

| Figure 1: Distribution of HRs estimated under an assumed HR of 0.60 for the transition hazards of the illness-death model in the simulation with fixed-effects data-generating mechanism, fixed-effects meta-analysis, and rank-preserving structural failure time models. The dashed line indicates the true value of the treatment policy estimand.  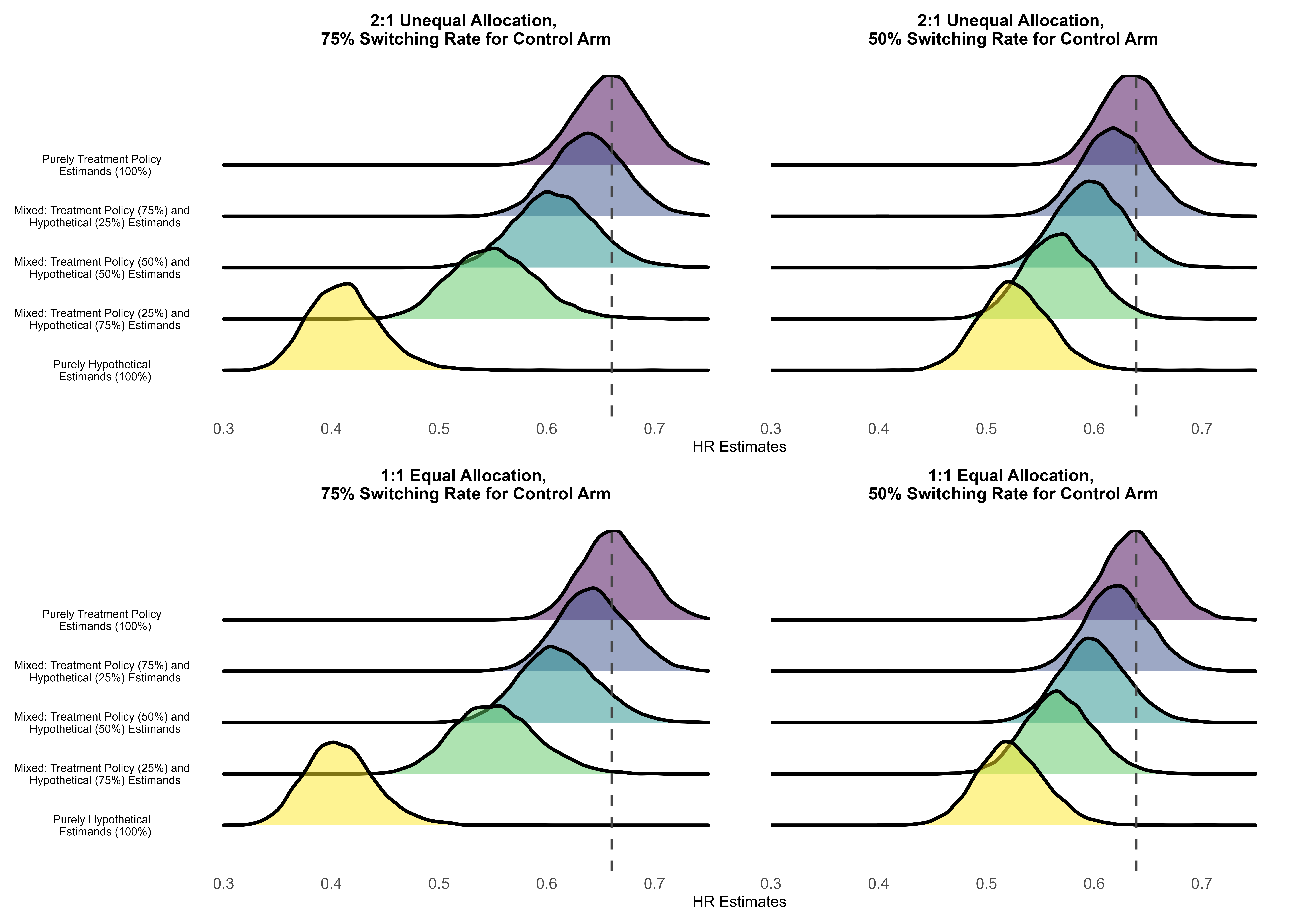 |
| --- |

| Figure 2: Distribution of HRs estimated under an assumed HR of 0.80 for the transition hazards of the illness-death model in the simulation with fixed-effects data-generating mechanism, fixed-effects meta-analysis, and rank-preserving structural failure time models. The dashed line indicates the true value of the treatment policy estimand.  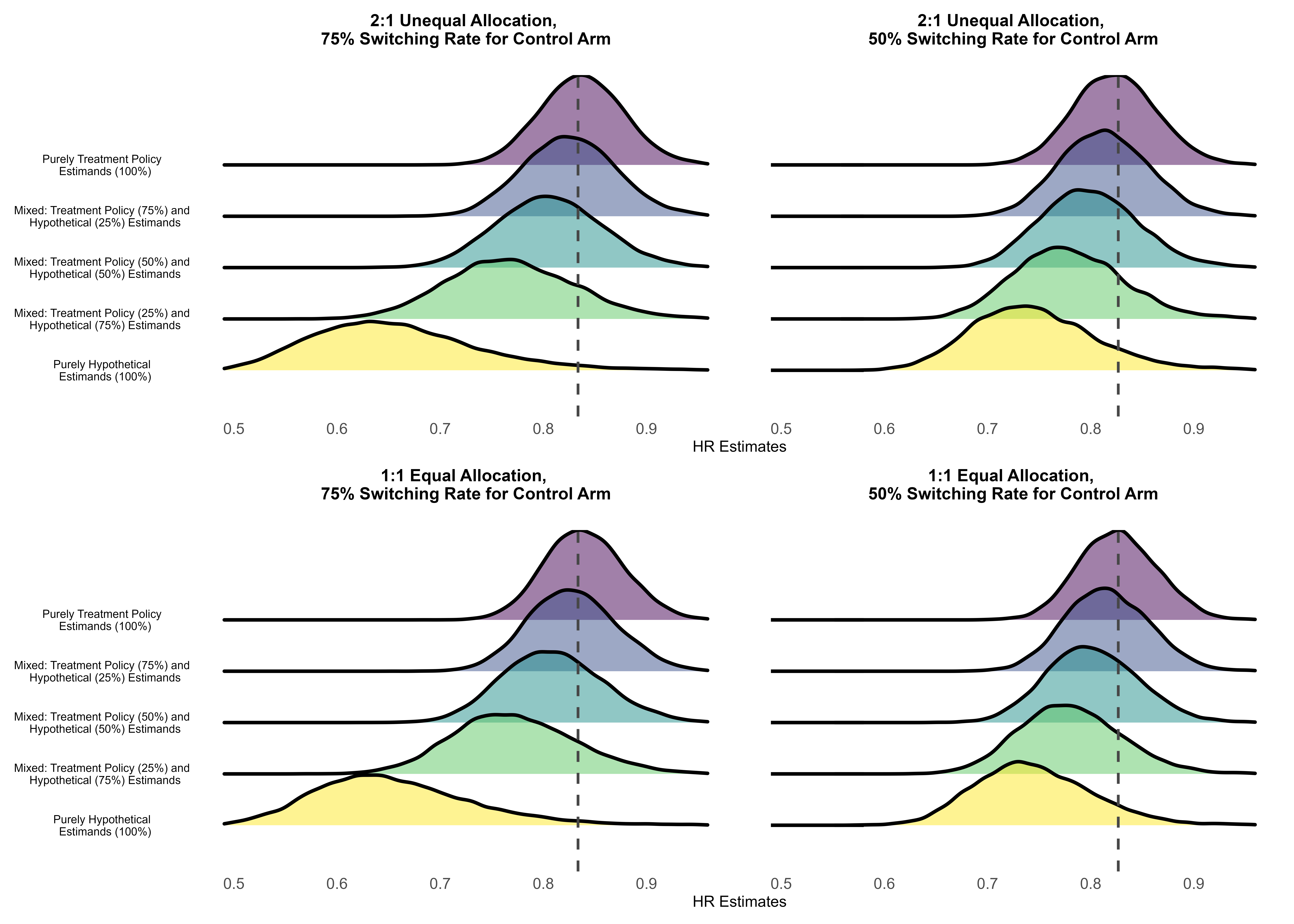 |
| --- |

| Figure 3: Distribution of HRs estimated under an assumed HR of 1.00 for the transition hazards of the illness-death model in the simulation with fixed-effects data-generating mechanism, fixed-effects meta-analysis, and rank-preserving structural failure time models. The dashed line indicates the true value of the treatment policy estimand.  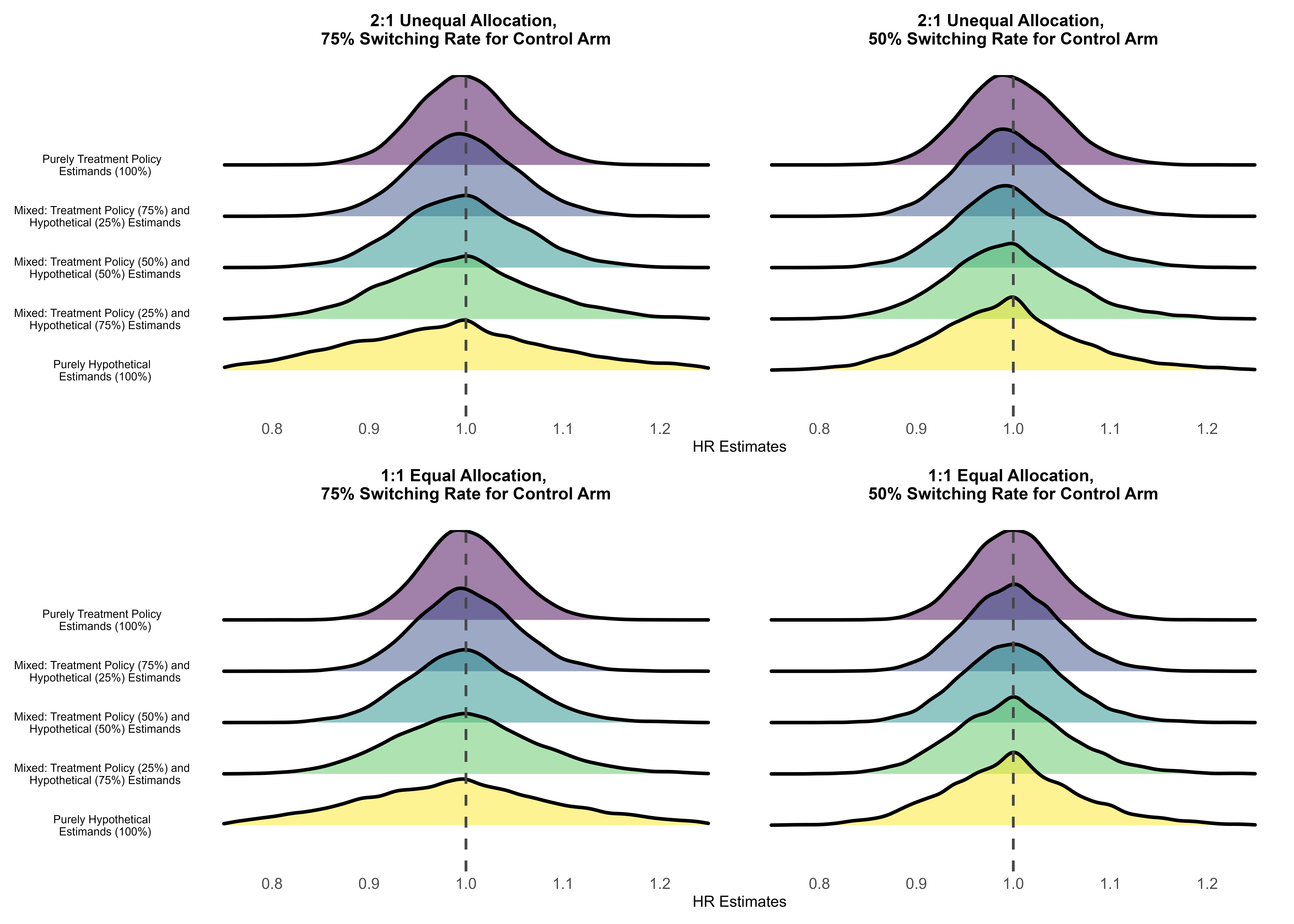 |
| --- |

## 2.2 Random-effects data-generating mechanism, random-effects meta-analysis

In this section, we present the results of the sensitivity analysis of the main simulation setting, where trials were simulated assuming a random-effects model within each simulation replicate. Here, for $n$ trials in a replicate under a scenario where the transition HR was $\beta$, we sampled $n$ study-specific log treatment effects $\log\beta_{1},...,log\beta_{n}$ from $N\left( \log\beta,\tau^{2} \right)$ for a pre-selected $\tau^{2}$ of 0.03. Then the individual trials were simulated as above using each $\beta_{i}$ as the specified transition HR. The sample size in each trial was randomly chosen to be 250, 300, or 350 with equal probability. For each scenario, we repeated the simulation 10,000 times, corresponding to 80,000 simulated trials. The trial estimates were pooled assuming a random-effects model.

| Table 4: Averages of pooled treatment effect estimates and comparison against treatment policy estimand under an assumed HR of 0.60 for the transition hazards of the illness-death model in the simulation with random-effects data-generating mechanism, random-effects meta-analysis, and rank-preserving structural failure time models   \| **Scenarios** \| **Estimators** \| **Estimated treatment effects:  HR (averaged 95% CI)** \| **Bias  (2.5%, 97.5% difference percentiles)** \| **Coverage** \| \| --- \| --- \| --- \| --- \| --- \| \| **75% switching rate for control arm** \|  \|  \| **Comparison against treatment policy estimand (True HR = 0.66)** \| \| \| 2:1 allocation,  75% switching rate for control arm^a^ \| Pure HE (100%) \| 0.44 (0.34, 0.57) \| -0.22 (-0.31, -0.09) \| 0.14 \| \| Mixed TPE (25%) and HE (75%) \| 0.53 (0.41, 0.68) \| -0.13 (-0.23, -0.01) \| 0.54 \| \| Mixed TPE (50%) and HE (50%) \| 0.59 (0.48, 0.72) \| -0.07 (-0.17, 0.04) \| 0.81 \| \| Mixed TPE (75%) and HE (25%) \| 0.63 (0.54, 0.74) \| -0.03 (-0.12, 0.08) \| 0.90 \| \| Pure TPE (100%) \| 0.66 (0.58, 0.75) \| -0.00 (-0.09, 0.10) \| 0.92 \| \| 1:1 allocation,  75% switching rate for control arm \| Pure HE (100%) \| 0.44 (0.34, 0.56) \| -0.22 (-0.31, -0.10) \| 0.13 \| \| Mixed TPE (25%) and HE (75%) \| 0.52 (0.40, 0.67) \| -0.14 (-0.23, -0.01) \| 0.52 \| \| Mixed TPE (50%) and HE (50%) \| 0.58 (0.47, 0.72) \| -0.08 (-0.17, 0.05) \| 0.81 \| \| Mixed TPE (75%) and HE (25%) \| 0.63 (0.54, 0.74) \| -0.03 (-0.12, 0.08) \| 0.91 \| \| Pure TPE (100%) \| 0.66 (0.58, 0.75) \| 0.00 (-0.08, 0.10) \| 0.92 \| \| **50% switching rate for control arm** \|  \|  \| **Comparison against treatment policy estimand (True HR = 0.64)** \| \| \| 2:1 allocation,  50% switching rate for control arm \| Pure HE (100%) \| 0.53 (0.44, 0.63) \| -0.11 (-0.20, -0.00) \| 0.41 \| \| Mixed TPE (25%) and HE (75%) \| 0.56 (0.47, 0.67) \| -0.08 (-0.17, 0.03) \| 0.65 \| \| Mixed TPE (50%) and HE (50%) \| 0.59 (0.50, 0.70) \| -0.05 (-0.14, 0.05) \| 0.82 \| \| Mixed TPE (75%) and HE (25%) \| 0.61 (0.53, 0.72) \| -0.03 (-0.11, 0.08) \| 0.89 \| \| Pure TPE (100%) \| 0.64 (0.55, 0.73) \| -0.00 (-0.09, 0.09) \| 0.92 \| \| 1:1 allocation,  50% switching rate for control arm \| Pure HE (100%) \| 0.53 (0.44, 0.63) \| -0.11 (-0.20, -0.01) \| 0.39 \| \| Mixed TPE (25%) and HE (75%) \| 0.56 (0.47, 0.67) \| -0.08 (-0.17, 0.02) \| 0.65 \| \| Mixed TPE (50%) and HE (50%) \| 0.59 (0.50, 0.70) \| -0.05 (-0.14, 0.05) \| 0.82 \| \| Mixed TPE (75%) and HE (25%) \| 0.62 (0.53, 0.72) \| -0.02 (-0.11, 0.08) \| 0.90 \| \| Pure TPE (100%) \| 0.64 (0.56, 0.73) \| 0.00 (-0.09, 0.10) \| 0.92 \| \| **^a^This table shows estimated treatment effects under an assumed hazard ratio (HR) of 0.60 for the transition hazards of the illness-death model and bias and coverage in comparison to the true treatment policy estimand. Monte Carlo standard errors for all measures are very close to zero  Acronyms: CI: Confidence intervals; HE - Hypothetical estimator; HR - Hazard ratio; TPE - Treatment policy estimator.** \| \| \| \| \| |
| --- | --- | --- | --- | --- | --- | --- | --- | --- | --- | --- | --- | --- | --- | --- | --- | --- | --- | --- | --- | --- | --- | --- | --- | --- | --- | --- | --- | --- | --- | --- | --- | --- | --- | --- | --- | --- | --- | --- | --- | --- | --- | --- | --- | --- | --- | --- | --- | --- | --- | --- | --- | --- | --- | --- | --- | --- | --- | --- | --- | --- | --- | --- | --- | --- | --- | --- | --- | --- | --- | --- | --- | --- | --- | --- | --- | --- | --- | --- | --- | --- | --- | --- | --- | --- | --- | --- | --- | --- | --- | --- | --- | --- | --- | --- | --- | --- | --- | --- | --- | --- | --- | --- | --- | --- |

| Table 5: Averages of pooled treatment effect estimates and comparison against treatment policy estimand under an assumed HR of 0.80 for the transition hazards of the illness-death model in the simulation with random-effects data-generating mechanism, random-effects meta-analysis, and rank-preserving structural failure time models   \| **Scenarios** \| **Estimators** \| **Estimated treatment effects:  HR (averaged 95% CI)** \| **Bias  (2.5%, 97.5% difference percentiles)** \| **Coverage** \| \| --- \| --- \| --- \| --- \| --- \| \| **75% switching rate for control arm** \|  \|  \| **Comparison against treatment policy estimand (True HR = 0.84)** \| \| \| 2:1 allocation,  75% switching rate for control arm^a^ \| Pure HE (100%) \| 0.66 (0.49, 0.89) \| -0.18 (-0.35, 0.06) \| 0.55 \| \| Mixed TPE (25%) and HE (75%) \| 0.73 (0.57, 0.93) \| -0.11 (-0.27, 0.08) \| 0.75 \| \| Mixed TPE (50%) and HE (50%) \| 0.78 (0.64, 0.95) \| -0.06 (-0.20, 0.10) \| 0.86 \| \| Mixed TPE (75%) and HE (25%) \| 0.81 (0.70, 0.95) \| -0.03 (-0.15, 0.11) \| 0.91 \| \| Pure TPE (100%) \| 0.84 (0.73, 0.96) \| -0.00 (-0.11, 0.12) \| 0.92 \| \| 1:1 allocation,  75% switching rate for control arm \| Pure HE (100%) \| 0.66 (0.49, 0.89) \| -0.18 (-0.35, 0.05) \| 0.56 \| \| Mixed TPE (25%) and HE (75%) \| 0.73 (0.57, 0.93) \| -0.11 (-0.27, 0.08) \| 0.76 \| \| Mixed TPE (50%) and HE (50%) \| 0.78 (0.64, 0.95) \| -0.06 (-0.20, 0.10) \| 0.87 \| \| Mixed TPE (75%) and HE (25%) \| 0.82 (0.70, 0.95) \| -0.02 (-0.14, 0.11) \| 0.91 \| \| Pure TPE (100%) \| 0.84 (0.74, 0.95) \| -0.00 (-0.10, 0.11) \| 0.92 \| \| **50% switching rate for control arm** \|  \|  \| **Comparison against treatment policy estimand (True HR = 0.83)** \| \| \| 2:1 allocation,  50% switching rate for control arm \| Pure HE (100%) \| 0.74 (0.61, 0.91) \| -0.08 (-0.23, 0.09) \| 0.73 \| \| Mixed TPE (25%) and HE (75%) \| 0.77 (0.64, 0.93) \| -0.06 (-0.19, 0.10) \| 0.81 \| \| Mixed TPE (50%) and HE (50%) \| 0.79 (0.67, 0.94) \| -0.04 (-0.16, 0.11) \| 0.87 \| \| Mixed TPE (75%) and HE (25%) \| 0.81 (0.69, 0.94) \| -0.02 (-0.14, 0.12) \| 0.90 \| \| Pure TPE (100%) \| 0.82 (0.72, 0.95) \| -0.00 (-0.11, 0.12) \| 0.92 \| \| 1:1 allocation,  50% switching rate for control arm \| Pure HE (100%) \| 0.74 (0.61, 0.91) \| -0.08 (-0.22, 0.09) \| 0.72 \| \| Mixed TPE (25%) and HE (75%) \| 0.77 (0.64, 0.93) \| -0.06 (-0.19, 0.10) \| 0.81 \| \| Mixed TPE (50%) and HE (50%) \| 0.79 (0.67, 0.94) \| -0.03 (-0.16, 0.11) \| 0.87 \| \| Mixed TPE (75%) and HE (25%) \| 0.81 (0.70, 0.94) \| -0.02 (-0.13, 0.11) \| 0.90 \| \| Pure TPE (100%) \| 0.82 (0.72, 0.95) \| -0.00 (-0.11, 0.12) \| 0.92 \| \| **^a^This table shows estimated treatment effects under an assumed hazard ratio (HR) of 0.80 for the transition hazards of the illness-death model and bias and coverage in comparison to the true treatment policy estimand. Monte Carlo standard errors for all measures are very close to zero  Acronyms: CI: Confidence intervals; HE - Hypothetical estimator; HR - Hazard ratio; TPE - Treatment policy estimator.** \| \| \| \| \| |
| --- | --- | --- | --- | --- | --- | --- | --- | --- | --- | --- | --- | --- | --- | --- | --- | --- | --- | --- | --- | --- | --- | --- | --- | --- | --- | --- | --- | --- | --- | --- | --- | --- | --- | --- | --- | --- | --- | --- | --- | --- | --- | --- | --- | --- | --- | --- | --- | --- | --- | --- | --- | --- | --- | --- | --- | --- | --- | --- | --- | --- | --- | --- | --- | --- | --- | --- | --- | --- | --- | --- | --- | --- | --- | --- | --- | --- | --- | --- | --- | --- | --- | --- | --- | --- | --- | --- | --- | --- | --- | --- | --- | --- | --- | --- | --- | --- | --- | --- | --- | --- | --- | --- | --- | --- |

| Table 6: Averages of pooled treatment effect estimates and comparison against treatment policy estimand under an assumed HR of 1.00 for the transition hazards of the illness-death model in the simulation with random-effects data-generating mechanism, random-effects meta-analysis, and rank-preserving structural failure time models   \| **Scenarios** \| **Estimators** \| **Estimated treatment effects:  HR (averaged 95% CI)** \| **Bias  (2.5%, 97.5% difference percentiles)** \| **Coverage** \| \| --- \| --- \| --- \| --- \| --- \| \| **75% switching rate for control arm** \|  \|  \| **Comparison against treatment policy estimand (True HR = 1.00)** \| \| \| 2:1 allocation,  75% switching rate for control arm^a^ \| Pure HE (100%) \| 0.98 (0.72, 1.35) \| -0.02 (-0.30, 0.34) \| 0.87 \| \| Mixed TPE (25%) and HE (75%) \| 0.98 (0.78, 1.25) \| -0.02 (-0.23, 0.24) \| 0.88 \| \| Mixed TPE (50%) and HE (50%) \| 0.99 (0.82, 1.19) \| -0.01 (-0.18, 0.18) \| 0.90 \| \| Mixed TPE (75%) and HE (25%) \| 0.99 (0.85, 1.16) \| -0.01 (-0.15, 0.15) \| 0.91 \| \| Pure TPE (100%) \| 1.00 (0.87, 1.14) \| -0.00 (-0.13, 0.13) \| 0.92 \| \| 1:1 allocation,  75% switching rate for control arm \| Pure HE (100%) \| 0.99 (0.73, 1.35) \| -0.01 (-0.29, 0.35) \| 0.87 \| \| Mixed TPE (25%) and HE (75%) \| 0.99 (0.79, 1.25) \| -0.01 (-0.22, 0.25) \| 0.89 \| \| Mixed TPE (50%) and HE (50%) \| 0.99 (0.83, 1.19) \| -0.01 (-0.18, 0.19) \| 0.90 \| \| Mixed TPE (75%) and HE (25%) \| 1.00 (0.86, 1.16) \| -0.00 (-0.14, 0.16) \| 0.91 \| \| Pure TPE (100%) \| 1.00 (0.88, 1.14) \| -0.00 (-0.12, 0.13) \| 0.92 \| \| **50% switching rate for control arm** \|  \|  \| **Comparison against treatment policy estimand (True HR = 1.00)** \| \| \| 2:1 allocation,  50% switching rate for control arm \| Pure HE (100%) \| 1.00 (0.80, 1.24) \| -0.00 (-0.21, 0.25) \| 0.85 \| \| Mixed TPE (25%) and HE (75%) \| 1.00 (0.82, 1.21) \| -0.00 (-0.18, 0.21) \| 0.87 \| \| Mixed TPE (50%) and HE (50%) \| 1.00 (0.84, 1.18) \| -0.00 (-0.17, 0.18) \| 0.89 \| \| Mixed TPE (75%) and HE (25%) \| 1.00 (0.86, 1.16) \| -0.00 (-0.15, 0.16) \| 0.90 \| \| Pure TPE (100%) \| 1.00 (0.87, 1.15) \| -0.00 (-0.14, 0.14) \| 0.92 \| \| 1:1 allocation,  50% switching rate for control arm \| Pure HE (100%) \| 1.00 (0.81, 1.25) \| 0.00 (-0.20, 0.25) \| 0.86 \| \| Mixed TPE (25%) and HE (75%) \| 1.00 (0.83, 1.21) \| 0.00 (-0.18, 0.21) \| 0.87 \| \| Mixed TPE (50%) and HE (50%) \| 1.00 (0.85, 1.18) \| 0.00 (-0.16, 0.18) \| 0.89 \| \| Mixed TPE (75%) and HE (25%) \| 1.00 (0.86, 1.16) \| -0.00 (-0.14, 0.16) \| 0.90 \| \| Pure TPE (100%) \| 1.00 (0.87, 1.14) \| -0.00 (-0.13, 0.14) \| 0.92 \| \| **^a^This table shows estimated treatment effects under an assumed hazard ratio (HR) of 1.00 for the transition hazards of the illness-death model and bias and coverage in comparison to the true treatment policy estimand. Monte Carlo standard errors for all measures are very close to zero  Acronyms: CI: Confidence intervals; HE - Hypothetical estimator; HR - Hazard ratio; TPE - Treatment policy estimator.** \| \| \| \| \| |
| --- | --- | --- | --- | --- | --- | --- | --- | --- | --- | --- | --- | --- | --- | --- | --- | --- | --- | --- | --- | --- | --- | --- | --- | --- | --- | --- | --- | --- | --- | --- | --- | --- | --- | --- | --- | --- | --- | --- | --- | --- | --- | --- | --- | --- | --- | --- | --- | --- | --- | --- | --- | --- | --- | --- | --- | --- | --- | --- | --- | --- | --- | --- | --- | --- | --- | --- | --- | --- | --- | --- | --- | --- | --- | --- | --- | --- | --- | --- | --- | --- | --- | --- | --- | --- | --- | --- | --- | --- | --- | --- | --- | --- | --- | --- | --- | --- | --- | --- | --- | --- | --- | --- | --- | --- |

| Figure 4: Distribution of HRs estimated under an assumed HR of 0.60 for the transition hazards of the illness-death model in the simulation with random-effects data-generating mechanism, random-effects meta-analysis, and rank-preserving structural failure time models. The dashed line indicates the true value of the treatment policy estimand.  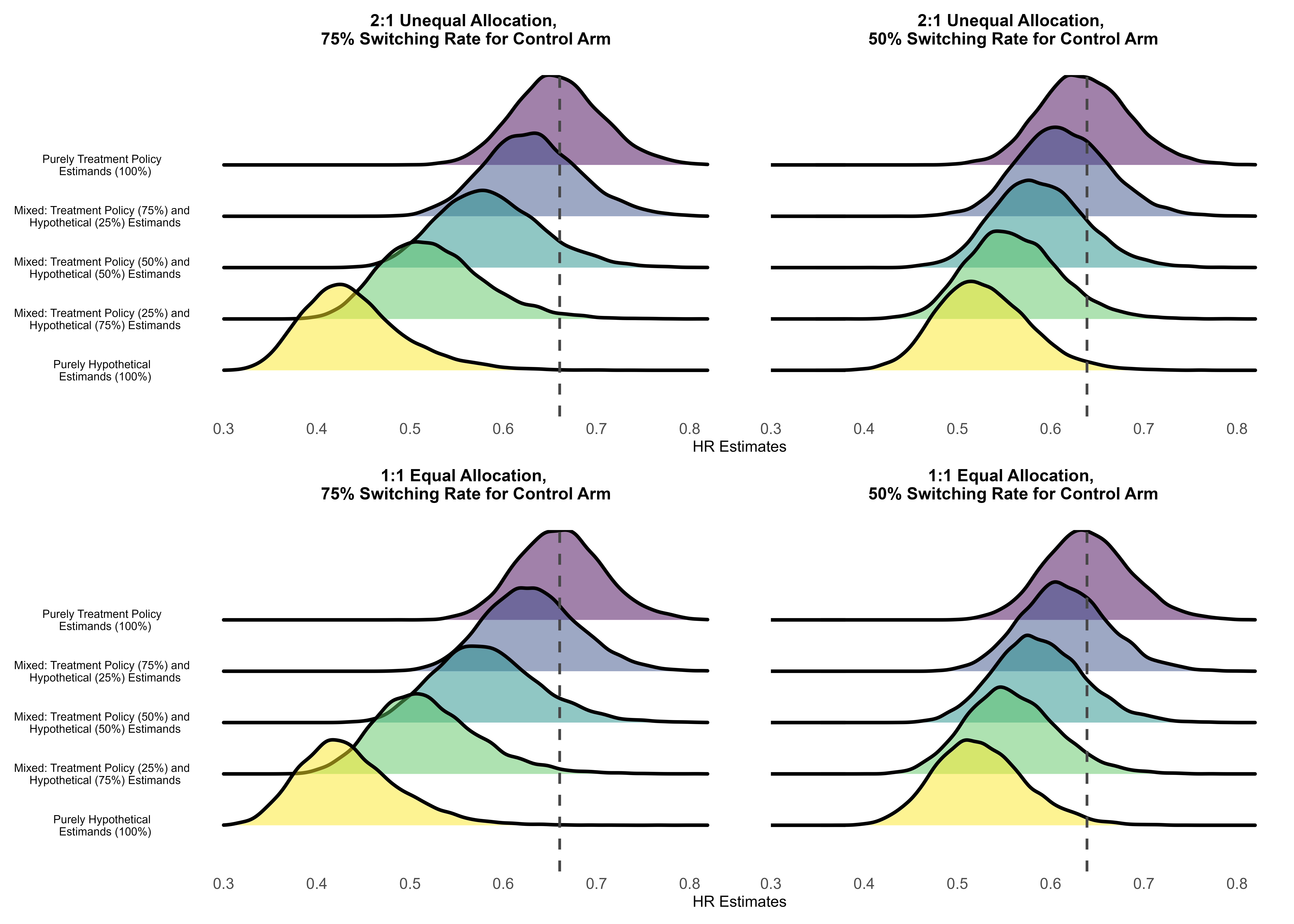 |
| --- |

| Figure 5: Distribution of HRs estimated under an assumed HR of 0.80 for the transition hazards of the illness-death model in the simulation with random-effects data-generating mechanism, random-effects meta-analysis, and rank-preserving structural failure time models. The dashed line indicates the true value of the treatment policy estimand.  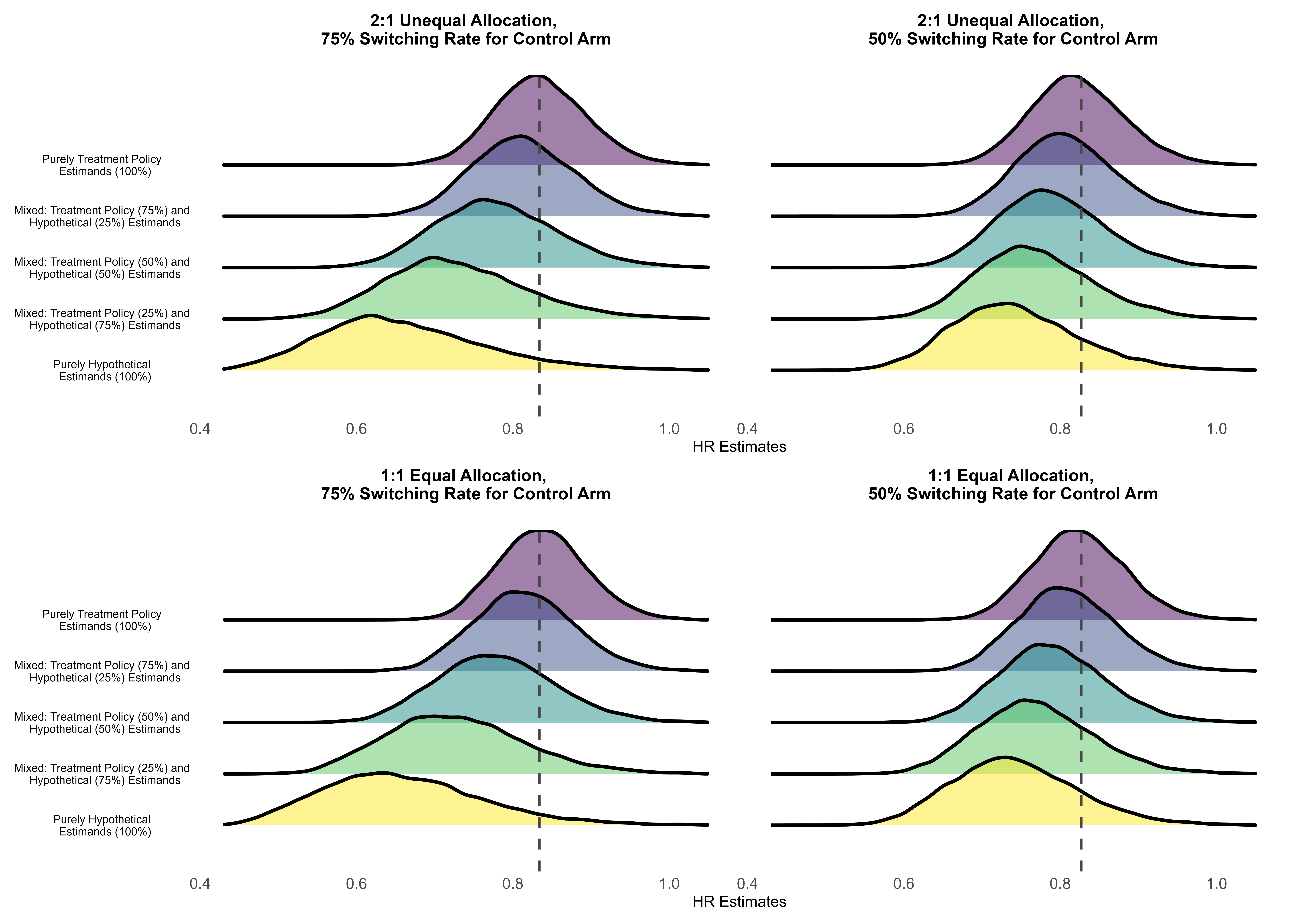 |
| --- |

| Figure 6: Distribution of HRs estimated under an assumed HR of 1.00 for the transition hazards of the illness-death model in the simulation with random-effects data-generating mechanism, random-effects meta-analysis, and rank-preserving structural failure time models. The dashed line indicates the true value of the treatment policy estimand.  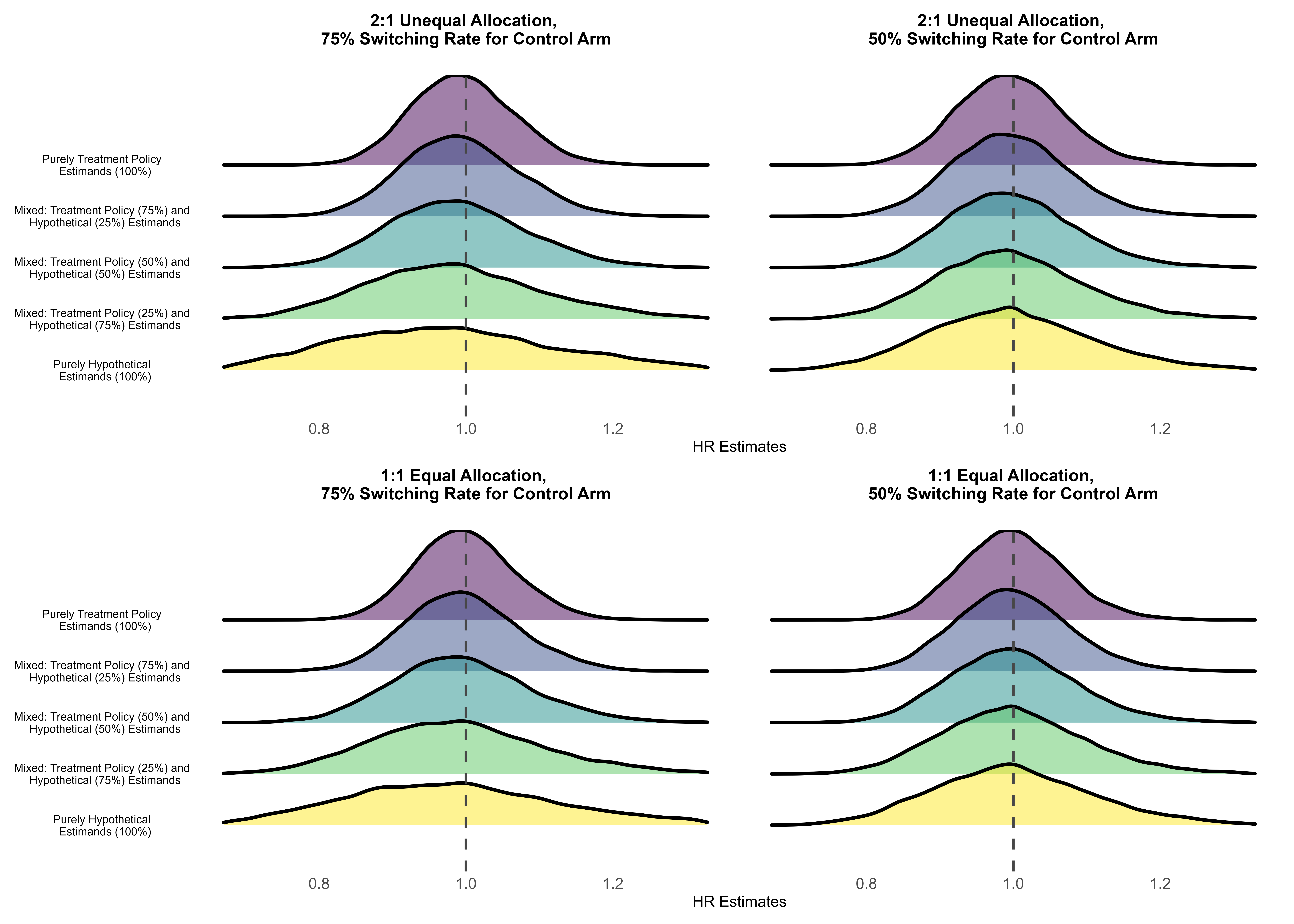 |
| --- |

# 3. Supplementary simulations

In this section, we present the results of the supplementary simulation setting, where the analytical strategy to target the hypothetical estimand was censoring switchers instead of rank-preserving structural failure time models. The data-generating mechanism and meta-analysis model of each supplementary simulation setting were identical to the corresponding main simulation.

## 3.1 Fixed-effects data-generating mechanism, random-effects meta-analysis

| Table 7: Averages of pooled treatment effect estimates and comparison against treatment policy estimand under an assumed HR of 0.60 for the transition hazards of the illness-death model in the simulation with fixed-effects data-generating mechanism, random-effects meta-analysis, and censoring switchers   \| **Scenarios** \| **Estimators** \| **Estimated treatment effects:  HR (averaged 95% CI)** \| **Bias  (2.5%, 97.5% difference percentiles)** \| **Coverage** \| \| --- \| --- \| --- \| --- \| --- \| \| **75% switching rate for control arm** \|  \|  \| **Comparison against treatment policy estimand (True HR = 0.66)** \| \| \| 2:1 allocation,  75% switching rate for control arm^a^ \| Pure HE (100%) \| 0.61 (0.51, 0.72) \| -0.05 (-0.14, 0.05) \| 0.85 \| \| Mixed TPE (25%) and HE (75%) \| 0.63 (0.54, 0.73) \| -0.03 (-0.11, 0.05) \| 0.91 \| \| Mixed TPE (50%) and HE (50%) \| 0.64 (0.57, 0.73) \| -0.02 (-0.09, 0.06) \| 0.95 \| \| Mixed TPE (75%) and HE (25%) \| 0.65 (0.58, 0.73) \| -0.01 (-0.07, 0.06) \| 0.96 \| \| Pure TPE (100%) \| 0.66 (0.60, 0.73) \| 0.00 (-0.06, 0.06) \| 0.96 \| \| 1:1 allocation,  75% switching rate for control arm \| Pure HE (100%) \| 0.61 (0.52, 0.72) \| -0.05 (-0.13, 0.04) \| 0.85 \| \| Mixed TPE (25%) and HE (75%) \| 0.63 (0.55, 0.72) \| -0.03 (-0.10, 0.05) \| 0.92 \| \| Mixed TPE (50%) and HE (50%) \| 0.65 (0.57, 0.73) \| -0.01 (-0.08, 0.06) \| 0.95 \| \| Mixed TPE (75%) and HE (25%) \| 0.65 (0.59, 0.73) \| -0.00 (-0.07, 0.06) \| 0.96 \| \| Pure TPE (100%) \| 0.66 (0.60, 0.73) \| 0.00 (-0.05, 0.06) \| 0.96 \| \| **50% switching rate for control arm** \|  \|  \| **Comparison against treatment policy estimand (True HR = 0.64)** \| \| \| 2:1 allocation,  50% switching rate for control arm \| Pure HE (100%) \| 0.60 (0.53, 0.69) \| -0.04 (-0.11, 0.04) \| 0.86 \| \| Mixed TPE (25%) and HE (75%) \| 0.61 (0.54, 0.69) \| -0.03 (-0.09, 0.04) \| 0.91 \| \| Mixed TPE (50%) and HE (50%) \| 0.62 (0.55, 0.70) \| -0.02 (-0.08, 0.05) \| 0.94 \| \| Mixed TPE (75%) and HE (25%) \| 0.63 (0.57, 0.70) \| -0.01 (-0.07, 0.06) \| 0.95 \| \| Pure TPE (100%) \| 0.64 (0.58, 0.71) \| -0.00 (-0.06, 0.06) \| 0.96 \| \| 1:1 allocation,  50% switching rate for control arm \| Pure HE (100%) \| 0.60 (0.53, 0.68) \| -0.04 (-0.10, 0.03) \| 0.86 \| \| Mixed TPE (25%) and HE (75%) \| 0.61 (0.55, 0.69) \| -0.02 (-0.09, 0.04) \| 0.91 \| \| Mixed TPE (50%) and HE (50%) \| 0.62 (0.56, 0.70) \| -0.01 (-0.07, 0.05) \| 0.94 \| \| Mixed TPE (75%) and HE (25%) \| 0.63 (0.57, 0.70) \| -0.01 (-0.06, 0.06) \| 0.96 \| \| Pure TPE (100%) \| 0.64 (0.58, 0.71) \| 0.00 (-0.06, 0.06) \| 0.96 \| \| **^a^This table shows estimated treatment effects under an assumed hazard ratio (HR) of 0.60 for the transition hazards of the illness-death model and bias and coverage in comparison to the true treatment policy estimand. Monte Carlo standard errors for all measures are very close to zero  Acronyms: CI: Confidence intervals; HE - Hypothetical estimator; HR - Hazard ratio; TPE - Treatment policy estimator.** \| \| \| \| \| |
| --- | --- | --- | --- | --- | --- | --- | --- | --- | --- | --- | --- | --- | --- | --- | --- | --- | --- | --- | --- | --- | --- | --- | --- | --- | --- | --- | --- | --- | --- | --- | --- | --- | --- | --- | --- | --- | --- | --- | --- | --- | --- | --- | --- | --- | --- | --- | --- | --- | --- | --- | --- | --- | --- | --- | --- | --- | --- | --- | --- | --- | --- | --- | --- | --- | --- | --- | --- | --- | --- | --- | --- | --- | --- | --- | --- | --- | --- | --- | --- | --- | --- | --- | --- | --- | --- | --- | --- | --- | --- | --- | --- | --- | --- | --- | --- | --- | --- | --- | --- | --- | --- | --- | --- | --- |

| Table 8: Averages of pooled treatment effect estimates and comparison against treatment policy estimand under an assumed HR of 0.80 for the transition hazards of the illness-death model in the simulation with fixed-effects data-generating mechanism, random-effects meta-analysis, and censoring switchers   \| **Scenarios** \| **Estimators** \| **Estimated treatment effects:  HR (averaged 95% CI)** \| **Bias  (2.5%, 97.5% difference percentiles)** \| **Coverage** \| \| --- \| --- \| --- \| --- \| --- \| \| **75% switching rate for control arm** \|  \|  \| **Comparison against treatment policy estimand (True HR = 0.84)** \| \| \| 2:1 allocation,  75% switching rate for control arm^a^ \| Pure HE (100%) \| 0.81 (0.68, 0.97) \| -0.03 (-0.15, 0.10) \| 0.94 \| \| Mixed TPE (25%) and HE (75%) \| 0.82 (0.71, 0.95) \| -0.02 (-0.12, 0.10) \| 0.95 \| \| Mixed TPE (50%) and HE (50%) \| 0.83 (0.73, 0.94) \| -0.01 (-0.10, 0.09) \| 0.96 \| \| Mixed TPE (75%) and HE (25%) \| 0.83 (0.74, 0.93) \| -0.01 (-0.09, 0.08) \| 0.96 \| \| Pure TPE (100%) \| 0.84 (0.76, 0.93) \| -0.00 (-0.08, 0.08) \| 0.96 \| \| 1:1 allocation,  75% switching rate for control arm \| Pure HE (100%) \| 0.81 (0.69, 0.96) \| -0.03 (-0.14, 0.10) \| 0.94 \| \| Mixed TPE (25%) and HE (75%) \| 0.82 (0.72, 0.95) \| -0.02 (-0.12, 0.09) \| 0.95 \| \| Mixed TPE (50%) and HE (50%) \| 0.83 (0.74, 0.94) \| -0.01 (-0.10, 0.09) \| 0.96 \| \| Mixed TPE (75%) and HE (25%) \| 0.84 (0.75, 0.93) \| -0.00 (-0.08, 0.08) \| 0.96 \| \| Pure TPE (100%) \| 0.84 (0.76, 0.93) \| 0.00 (-0.07, 0.08) \| 0.96 \| \| **50% switching rate for control arm** \|  \|  \| **Comparison against treatment policy estimand (True HR = 0.83)** \| \| \| 2:1 allocation,  50% switching rate for control arm \| Pure HE (100%) \| 0.80 (0.70, 0.92) \| -0.02 (-0.12, 0.08) \| 0.93 \| \| Mixed TPE (25%) and HE (75%) \| 0.81 (0.71, 0.92) \| -0.02 (-0.11, 0.08) \| 0.95 \| \| Mixed TPE (50%) and HE (50%) \| 0.81 (0.73, 0.92) \| -0.01 (-0.10, 0.08) \| 0.95 \| \| Mixed TPE (75%) and HE (25%) \| 0.82 (0.73, 0.91) \| -0.01 (-0.09, 0.08) \| 0.96 \| \| Pure TPE (100%) \| 0.82 (0.74, 0.91) \| -0.00 (-0.08, 0.08) \| 0.96 \| \| 1:1 allocation,  50% switching rate for control arm \| Pure HE (100%) \| 0.80 (0.71, 0.91) \| -0.02 (-0.11, 0.07) \| 0.94 \| \| Mixed TPE (25%) and HE (75%) \| 0.81 (0.72, 0.91) \| -0.02 (-0.10, 0.08) \| 0.95 \| \| Mixed TPE (50%) and HE (50%) \| 0.82 (0.73, 0.91) \| -0.01 (-0.09, 0.08) \| 0.96 \| \| Mixed TPE (75%) and HE (25%) \| 0.82 (0.74, 0.91) \| -0.00 (-0.08, 0.08) \| 0.96 \| \| Pure TPE (100%) \| 0.83 (0.75, 0.91) \| 0.00 (-0.07, 0.08) \| 0.96 \| \| **^a^This table shows estimated treatment effects under an assumed hazard ratio (HR) of 0.80 for the transition hazards of the illness-death model and bias and coverage in comparison to the true treatment policy estimand. Monte Carlo standard errors for all measures are very close to zero  Acronyms: CI: Confidence intervals; HE - Hypothetical estimator; HR - Hazard ratio; TPE - Treatment policy estimator.** \| \| \| \| \| |
| --- | --- | --- | --- | --- | --- | --- | --- | --- | --- | --- | --- | --- | --- | --- | --- | --- | --- | --- | --- | --- | --- | --- | --- | --- | --- | --- | --- | --- | --- | --- | --- | --- | --- | --- | --- | --- | --- | --- | --- | --- | --- | --- | --- | --- | --- | --- | --- | --- | --- | --- | --- | --- | --- | --- | --- | --- | --- | --- | --- | --- | --- | --- | --- | --- | --- | --- | --- | --- | --- | --- | --- | --- | --- | --- | --- | --- | --- | --- | --- | --- | --- | --- | --- | --- | --- | --- | --- | --- | --- | --- | --- | --- | --- | --- | --- | --- | --- | --- | --- | --- | --- | --- | --- | --- |

| Table 9: Averages of pooled treatment effect estimates and comparison against treatment policy estimand under an assumed HR of 1.00 for the transition hazards of the illness-death model in the simulation with fixed-effects data-generating mechanism, random-effects meta-analysis, and censoring switchers   \| **Scenarios** \| **Estimators** \| **Estimated treatment effects:  HR (averaged 95% CI)** \| **Bias  (2.5%, 97.5% difference percentiles)** \| **Coverage** \| \| --- \| --- \| --- \| --- \| --- \| \| **75% switching rate for control arm** \|  \|  \| **Comparison against treatment policy estimand (True HR = 1.00)** \| \| \| 2:1 allocation,  75% switching rate for control arm^a^ \| Pure HE (100%) \| 1.01 (0.84, 1.21) \| 0.01 (-0.14, 0.19) \| 0.97 \| \| Mixed TPE (25%) and HE (75%) \| 1.00 (0.87, 1.17) \| 0.00 (-0.13, 0.15) \| 0.96 \| \| Mixed TPE (50%) and HE (50%) \| 1.00 (0.88, 1.14) \| 0.00 (-0.11, 0.13) \| 0.96 \| \| Mixed TPE (75%) and HE (25%) \| 1.00 (0.89, 1.12) \| 0.00 (-0.10, 0.11) \| 0.96 \| \| Pure TPE (100%) \| 1.00 (0.90, 1.11) \| -0.00 (-0.09, 0.10) \| 0.96 \| \| 1:1 allocation,  75% switching rate for control arm \| Pure HE (100%) \| 1.02 (0.86, 1.20) \| 0.02 (-0.13, 0.18) \| 0.96 \| \| Mixed TPE (25%) and HE (75%) \| 1.01 (0.88, 1.16) \| 0.01 (-0.11, 0.15) \| 0.96 \| \| Mixed TPE (50%) and HE (50%) \| 1.01 (0.89, 1.14) \| 0.01 (-0.10, 0.12) \| 0.96 \| \| Mixed TPE (75%) and HE (25%) \| 1.00 (0.90, 1.12) \| 0.00 (-0.09, 0.11) \| 0.96 \| \| Pure TPE (100%) \| 1.00 (0.91, 1.11) \| 0.00 (-0.09, 0.10) \| 0.96 \| \| **50% switching rate for control arm** \|  \|  \| **Comparison against treatment policy estimand (True HR = 1.00)** \| \| \| 2:1 allocation,  50% switching rate for control arm \| Pure HE (100%) \| 1.00 (0.87, 1.15) \| 0.00 (-0.12, 0.13) \| 0.96 \| \| Mixed TPE (25%) and HE (75%) \| 1.00 (0.88, 1.14) \| 0.00 (-0.11, 0.12) \| 0.96 \| \| Mixed TPE (50%) and HE (50%) \| 1.00 (0.89, 1.13) \| -0.00 (-0.11, 0.11) \| 0.96 \| \| Mixed TPE (75%) and HE (25%) \| 1.00 (0.89, 1.12) \| -0.00 (-0.10, 0.11) \| 0.96 \| \| Pure TPE (100%) \| 1.00 (0.90, 1.11) \| -0.00 (-0.09, 0.10) \| 0.96 \| \| 1:1 allocation,  50% switching rate for control arm \| Pure HE (100%) \| 1.00 (0.88, 1.14) \| 0.00 (-0.11, 0.12) \| 0.96 \| \| Mixed TPE (25%) and HE (75%) \| 1.00 (0.89, 1.13) \| 0.00 (-0.10, 0.11) \| 0.97 \| \| Mixed TPE (50%) and HE (50%) \| 1.00 (0.90, 1.12) \| 0.00 (-0.10, 0.11) \| 0.96 \| \| Mixed TPE (75%) and HE (25%) \| 1.00 (0.90, 1.11) \| 0.00 (-0.09, 0.10) \| 0.96 \| \| Pure TPE (100%) \| 1.00 (0.91, 1.11) \| 0.00 (-0.09, 0.10) \| 0.96 \| \| **^a^This table shows estimated treatment effects under an assumed hazard ratio (HR) of 1.00 for the transition hazards of the illness-death model and bias and coverage in comparison to the true treatment policy estimand. Monte Carlo standard errors for all measures are very close to zero  Acronyms: CI: Confidence intervals; HE - Hypothetical estimator; HR - Hazard ratio; TPE - Treatment policy estimator.** \| \| \| \| \| |
| --- | --- | --- | --- | --- | --- | --- | --- | --- | --- | --- | --- | --- | --- | --- | --- | --- | --- | --- | --- | --- | --- | --- | --- | --- | --- | --- | --- | --- | --- | --- | --- | --- | --- | --- | --- | --- | --- | --- | --- | --- | --- | --- | --- | --- | --- | --- | --- | --- | --- | --- | --- | --- | --- | --- | --- | --- | --- | --- | --- | --- | --- | --- | --- | --- | --- | --- | --- | --- | --- | --- | --- | --- | --- | --- | --- | --- | --- | --- | --- | --- | --- | --- | --- | --- | --- | --- | --- | --- | --- | --- | --- | --- | --- | --- | --- | --- | --- | --- | --- | --- | --- | --- | --- | --- |

## 3.2 Fixed-effects data-generating mechanism, fixed-effects meta-analysis

| Table 10: Averages of pooled treatment effect estimates and comparison against treatment policy estimand under an assumed HR of 0.60 for the transition hazards of the illness-death model in the simulation with fixed-effects data-generating mechanism, fixed-effects meta-analysis, and censoring switchers   \| **Scenarios** \| **Estimators** \| **Estimated treatment effects:  HR (averaged 95% CI)** \| **Bias  (2.5%, 97.5% difference percentiles)** \| **Coverage** \| \| --- \| --- \| --- \| --- \| --- \| \| **75% switching rate for control arm** \|  \|  \| **Comparison against treatment policy estimand (True HR = 0.66)** \| \| \| 2:1 allocation,  75% switching rate for control arm^a^ \| Pure HE (100%) \| 0.61 (0.52, 0.71) \| -0.05 (-0.14, 0.04) \| 0.81 \| \| Mixed TPE (25%) and HE (75%) \| 0.63 (0.56, 0.72) \| -0.03 (-0.10, 0.06) \| 0.89 \| \| Mixed TPE (50%) and HE (50%) \| 0.65 (0.58, 0.72) \| -0.01 (-0.08, 0.06) \| 0.93 \| \| Mixed TPE (75%) and HE (25%) \| 0.65 (0.59, 0.72) \| -0.01 (-0.07, 0.06) \| 0.94 \| \| Pure TPE (100%) \| 0.66 (0.60, 0.72) \| 0.00 (-0.06, 0.06) \| 0.95 \| \| 1:1 allocation,  75% switching rate for control arm \| Pure HE (100%) \| 0.61 (0.53, 0.71) \| -0.05 (-0.13, 0.05) \| 0.82 \| \| Mixed TPE (25%) and HE (75%) \| 0.63 (0.56, 0.72) \| -0.02 (-0.10, 0.06) \| 0.90 \| \| Mixed TPE (50%) and HE (50%) \| 0.65 (0.58, 0.72) \| -0.01 (-0.08, 0.06) \| 0.94 \| \| Mixed TPE (75%) and HE (25%) \| 0.66 (0.60, 0.72) \| -0.00 (-0.06, 0.06) \| 0.95 \| \| Pure TPE (100%) \| 0.66 (0.61, 0.72) \| 0.00 (-0.05, 0.06) \| 0.95 \| \| **50% switching rate for control arm** \|  \|  \| **Comparison against treatment policy estimand (True HR = 0.64)** \| \| \| 2:1 allocation,  50% switching rate for control arm \| Pure HE (100%) \| 0.60 (0.53, 0.68) \| -0.04 (-0.11, 0.04) \| 0.82 \| \| Mixed TPE (25%) and HE (75%) \| 0.61 (0.55, 0.69) \| -0.03 (-0.09, 0.05) \| 0.89 \| \| Mixed TPE (50%) and HE (50%) \| 0.62 (0.56, 0.69) \| -0.02 (-0.08, 0.05) \| 0.92 \| \| Mixed TPE (75%) and HE (25%) \| 0.63 (0.57, 0.70) \| -0.01 (-0.07, 0.06) \| 0.94 \| \| Pure TPE (100%) \| 0.64 (0.58, 0.70) \| -0.00 (-0.06, 0.06) \| 0.95 \| \| 1:1 allocation,  50% switching rate for control arm \| Pure HE (100%) \| 0.60 (0.54, 0.68) \| -0.04 (-0.10, 0.03) \| 0.83 \| \| Mixed TPE (25%) and HE (75%) \| 0.62 (0.55, 0.68) \| -0.02 (-0.09, 0.04) \| 0.89 \| \| Mixed TPE (50%) and HE (50%) \| 0.63 (0.57, 0.69) \| -0.01 (-0.07, 0.05) \| 0.92 \| \| Mixed TPE (75%) and HE (25%) \| 0.63 (0.58, 0.70) \| -0.01 (-0.06, 0.06) \| 0.94 \| \| Pure TPE (100%) \| 0.64 (0.59, 0.70) \| 0.00 (-0.05, 0.06) \| 0.95 \| \| **^a^This table shows estimated treatment effects under an assumed hazard ratio (HR) of 0.60 for the transition hazards of the illness-death model and bias and coverage in comparison to the true treatment policy estimand. Monte Carlo standard errors for all measures are very close to zero  Acronyms: CI: Confidence intervals; HE - Hypothetical estimator; HR - Hazard ratio; TPE - Treatment policy estimator.** \| \| \| \| \| |
| --- | --- | --- | --- | --- | --- | --- | --- | --- | --- | --- | --- | --- | --- | --- | --- | --- | --- | --- | --- | --- | --- | --- | --- | --- | --- | --- | --- | --- | --- | --- | --- | --- | --- | --- | --- | --- | --- | --- | --- | --- | --- | --- | --- | --- | --- | --- | --- | --- | --- | --- | --- | --- | --- | --- | --- | --- | --- | --- | --- | --- | --- | --- | --- | --- | --- | --- | --- | --- | --- | --- | --- | --- | --- | --- | --- | --- | --- | --- | --- | --- | --- | --- | --- | --- | --- | --- | --- | --- | --- | --- | --- | --- | --- | --- | --- | --- | --- | --- | --- | --- | --- | --- | --- | --- |

| Table 11: Averages of pooled treatment effect estimates and comparison against treatment policy estimand under an assumed HR of 0.80 for the transition hazards of the illness-death model in the simulation with fixed-effects data-generating mechanism, fixed-effects meta-analysis, and censoring switchers   \| **Scenarios** \| **Estimators** \| **Estimated treatment effects:  HR (averaged 95% CI)** \| **Bias  (2.5%, 97.5% difference percentiles)** \| **Coverage** \| \| --- \| --- \| --- \| --- \| --- \| \| **75% switching rate for control arm** \|  \|  \| **Comparison against treatment policy estimand (True HR = 0.84)** \| \| \| 2:1 allocation,  75% switching rate for control arm^a^ \| Pure HE (100%) \| 0.81 (0.69, 0.95) \| -0.03 (-0.15, 0.10) \| 0.92 \| \| Mixed TPE (25%) and HE (75%) \| 0.82 (0.72, 0.94) \| -0.02 (-0.12, 0.10) \| 0.94 \| \| Mixed TPE (50%) and HE (50%) \| 0.83 (0.74, 0.93) \| -0.01 (-0.10, 0.09) \| 0.95 \| \| Mixed TPE (75%) and HE (25%) \| 0.83 (0.75, 0.93) \| -0.01 (-0.09, 0.08) \| 0.95 \| \| Pure TPE (100%) \| 0.84 (0.76, 0.92) \| -0.00 (-0.08, 0.08) \| 0.95 \| \| 1:1 allocation,  75% switching rate for control arm \| Pure HE (100%) \| 0.81 (0.70, 0.94) \| -0.03 (-0.14, 0.10) \| 0.93 \| \| Mixed TPE (25%) and HE (75%) \| 0.83 (0.73, 0.93) \| -0.01 (-0.11, 0.09) \| 0.94 \| \| Mixed TPE (50%) and HE (50%) \| 0.83 (0.75, 0.93) \| -0.01 (-0.09, 0.09) \| 0.95 \| \| Mixed TPE (75%) and HE (25%) \| 0.84 (0.76, 0.92) \| -0.00 (-0.08, 0.08) \| 0.95 \| \| Pure TPE (100%) \| 0.84 (0.77, 0.92) \| 0.00 (-0.07, 0.08) \| 0.95 \| \| **50% switching rate for control arm** \|  \|  \| **Comparison against treatment policy estimand (True HR = 0.83)** \| \| \| 2:1 allocation,  50% switching rate for control arm \| Pure HE (100%) \| 0.80 (0.71, 0.91) \| -0.03 (-0.12, 0.08) \| 0.92 \| \| Mixed TPE (25%) and HE (75%) \| 0.81 (0.72, 0.91) \| -0.02 (-0.11, 0.08) \| 0.93 \| \| Mixed TPE (50%) and HE (50%) \| 0.81 (0.73, 0.91) \| -0.01 (-0.10, 0.08) \| 0.94 \| \| Mixed TPE (75%) and HE (25%) \| 0.82 (0.74, 0.91) \| -0.01 (-0.09, 0.08) \| 0.94 \| \| Pure TPE (100%) \| 0.82 (0.75, 0.91) \| -0.00 (-0.08, 0.08) \| 0.95 \| \| 1:1 allocation,  50% switching rate for control arm \| Pure HE (100%) \| 0.80 (0.72, 0.90) \| -0.02 (-0.11, 0.07) \| 0.92 \| \| Mixed TPE (25%) and HE (75%) \| 0.81 (0.73, 0.90) \| -0.01 (-0.10, 0.08) \| 0.94 \| \| Mixed TPE (50%) and HE (50%) \| 0.82 (0.74, 0.90) \| -0.01 (-0.09, 0.08) \| 0.94 \| \| Mixed TPE (75%) and HE (25%) \| 0.82 (0.75, 0.90) \| -0.00 (-0.08, 0.08) \| 0.95 \| \| Pure TPE (100%) \| 0.83 (0.76, 0.90) \| 0.00 (-0.07, 0.08) \| 0.95 \| \| **^a^This table shows estimated treatment effects under an assumed hazard ratio (HR) of 0.80 for the transition hazards of the illness-death model and bias and coverage in comparison to the true treatment policy estimand. Monte Carlo standard errors for all measures are very close to zero  Acronyms: CI: Confidence intervals; HE - Hypothetical estimator; HR - Hazard ratio; TPE - Treatment policy estimator.** \| \| \| \| \| |
| --- | --- | --- | --- | --- | --- | --- | --- | --- | --- | --- | --- | --- | --- | --- | --- | --- | --- | --- | --- | --- | --- | --- | --- | --- | --- | --- | --- | --- | --- | --- | --- | --- | --- | --- | --- | --- | --- | --- | --- | --- | --- | --- | --- | --- | --- | --- | --- | --- | --- | --- | --- | --- | --- | --- | --- | --- | --- | --- | --- | --- | --- | --- | --- | --- | --- | --- | --- | --- | --- | --- | --- | --- | --- | --- | --- | --- | --- | --- | --- | --- | --- | --- | --- | --- | --- | --- | --- | --- | --- | --- | --- | --- | --- | --- | --- | --- | --- | --- | --- | --- | --- | --- | --- | --- |

| Table 12: Averages of pooled treatment effect estimates and comparison against treatment policy estimand under an assumed HR of 1.00 for the transition hazards of the illness-death model in the simulation with fixed-effects data-generating mechanism, fixed-effects meta-analysis, and censoring switchers   \| **Scenarios** \| **Estimators** \| **Estimated treatment effects:  HR (averaged 95% CI)** \| **Bias  (2.5%, 97.5% difference percentiles)** \| **Coverage** \| \| --- \| --- \| --- \| --- \| --- \| \| **75% switching rate for control arm** \|  \|  \| **Comparison against treatment policy estimand (True HR = 1.00)** \| \| \| 2:1 allocation,  75% switching rate for control arm^a^ \| Pure HE (100%) \| 1.01 (0.85, 1.19) \| 0.01 (-0.15, 0.18) \| 0.96 \| \| Mixed TPE (25%) and HE (75%) \| 1.00 (0.87, 1.15) \| 0.00 (-0.13, 0.14) \| 0.95 \| \| Mixed TPE (50%) and HE (50%) \| 1.00 (0.89, 1.13) \| 0.00 (-0.11, 0.12) \| 0.95 \| \| Mixed TPE (75%) and HE (25%) \| 1.00 (0.90, 1.11) \| -0.00 (-0.10, 0.11) \| 0.95 \| \| Pure TPE (100%) \| 1.00 (0.91, 1.10) \| -0.00 (-0.09, 0.10) \| 0.95 \| \| 1:1 allocation,  75% switching rate for control arm \| Pure HE (100%) \| 1.02 (0.87, 1.18) \| 0.02 (-0.13, 0.18) \| 0.95 \| \| Mixed TPE (25%) and HE (75%) \| 1.01 (0.89, 1.14) \| 0.01 (-0.11, 0.14) \| 0.95 \| \| Mixed TPE (50%) and HE (50%) \| 1.00 (0.90, 1.12) \| 0.00 (-0.10, 0.12) \| 0.95 \| \| Mixed TPE (75%) and HE (25%) \| 1.00 (0.91, 1.11) \| 0.00 (-0.09, 0.11) \| 0.95 \| \| Pure TPE (100%) \| 1.00 (0.91, 1.10) \| 0.00 (-0.09, 0.10) \| 0.95 \| \| **50% switching rate for control arm** \|  \|  \| **Comparison against treatment policy estimand (True HR = 1.00)** \| \| \| 2:1 allocation,  50% switching rate for control arm \| Pure HE (100%) \| 1.00 (0.88, 1.13) \| -0.00 (-0.12, 0.13) \| 0.95 \| \| Mixed TPE (25%) and HE (75%) \| 1.00 (0.89, 1.12) \| -0.00 (-0.11, 0.12) \| 0.95 \| \| Mixed TPE (50%) and HE (50%) \| 1.00 (0.90, 1.11) \| -0.00 (-0.11, 0.11) \| 0.95 \| \| Mixed TPE (75%) and HE (25%) \| 1.00 (0.90, 1.11) \| -0.00 (-0.10, 0.10) \| 0.95 \| \| Pure TPE (100%) \| 1.00 (0.91, 1.10) \| -0.00 (-0.09, 0.10) \| 0.95 \| \| 1:1 allocation,  50% switching rate for control arm \| Pure HE (100%) \| 1.00 (0.89, 1.13) \| 0.00 (-0.11, 0.12) \| 0.95 \| \| Mixed TPE (25%) and HE (75%) \| 1.00 (0.90, 1.12) \| 0.00 (-0.10, 0.11) \| 0.95 \| \| Mixed TPE (50%) and HE (50%) \| 1.00 (0.90, 1.11) \| 0.00 (-0.10, 0.11) \| 0.95 \| \| Mixed TPE (75%) and HE (25%) \| 1.00 (0.91, 1.10) \| 0.00 (-0.09, 0.10) \| 0.95 \| \| Pure TPE (100%) \| 1.00 (0.91, 1.10) \| 0.00 (-0.09, 0.10) \| 0.95 \| \| **^a^This table shows estimated treatment effects under an assumed hazard ratio (HR) of 1.00 for the transition hazards of the illness-death model and bias and coverage in comparison to the true treatment policy estimand. Monte Carlo standard errors for all measures are very close to zero  Acronyms: CI: Confidence intervals; HE - Hypothetical estimator; HR - Hazard ratio; TPE - Treatment policy estimator.** \| \| \| \| \| |
| --- | --- | --- | --- | --- | --- | --- | --- | --- | --- | --- | --- | --- | --- | --- | --- | --- | --- | --- | --- | --- | --- | --- | --- | --- | --- | --- | --- | --- | --- | --- | --- | --- | --- | --- | --- | --- | --- | --- | --- | --- | --- | --- | --- | --- | --- | --- | --- | --- | --- | --- | --- | --- | --- | --- | --- | --- | --- | --- | --- | --- | --- | --- | --- | --- | --- | --- | --- | --- | --- | --- | --- | --- | --- | --- | --- | --- | --- | --- | --- | --- | --- | --- | --- | --- | --- | --- | --- | --- | --- | --- | --- | --- | --- | --- | --- | --- | --- | --- | --- | --- | --- | --- | --- | --- |

## 3.3 Random-effects data-generating mechanism, random-effects meta-analysis

| Table 13: Averages of pooled treatment effect estimates and comparison against treatment policy estimand under an assumed HR of 0.60 for the transition hazards of the illness-death model in the simulation with random-effects data-generating mechanism, random-effects meta-analysis, and censoring switchers   \| **Scenarios** \| **Estimators** \| **Estimated treatment effects:  HR (averaged 95% CI)** \| **Bias  (2.5%, 97.5% difference percentiles)** \| **Coverage** \| \| --- \| --- \| --- \| --- \| --- \| \| **75% switching rate for control arm** \|  \|  \| **Comparison against treatment policy estimand (True HR = 0.66)** \| \| \| 2:1 allocation,  75% switching rate for control arm^a^ \| Pure HE (100%) \| 0.61 (0.50, 0.74) \| -0.05 (-0.16, 0.08) \| 0.85 \| \| Mixed TPE (25%) and HE (75%) \| 0.63 (0.53, 0.75) \| -0.03 (-0.14, 0.08) \| 0.89 \| \| Mixed TPE (50%) and HE (50%) \| 0.64 (0.55, 0.75) \| -0.02 (-0.12, 0.09) \| 0.91 \| \| Mixed TPE (75%) and HE (25%) \| 0.65 (0.56, 0.75) \| -0.01 (-0.10, 0.10) \| 0.92 \| \| Pure TPE (100%) \| 0.66 (0.58, 0.76) \| -0.00 (-0.09, 0.10) \| 0.92 \| \| 1:1 allocation,  75% switching rate for control arm \| Pure HE (100%) \| 0.61 (0.51, 0.74) \| -0.05 (-0.16, 0.08) \| 0.84 \| \| Mixed TPE (25%) and HE (75%) \| 0.63 (0.53, 0.75) \| -0.03 (-0.13, 0.08) \| 0.88 \| \| Mixed TPE (50%) and HE (50%) \| 0.64 (0.55, 0.75) \| -0.02 (-0.11, 0.09) \| 0.91 \| \| Mixed TPE (75%) and HE (25%) \| 0.65 (0.57, 0.75) \| -0.01 (-0.10, 0.09) \| 0.91 \| \| Pure TPE (100%) \| 0.66 (0.58, 0.75) \| 0.00 (-0.08, 0.10) \| 0.92 \| \| **50% switching rate for control arm** \|  \|  \| **Comparison against treatment policy estimand (True HR = 0.64)** \| \| \| 2:1 allocation,  50% switching rate for control arm \| Pure HE (100%) \| 0.60 (0.51, 0.71) \| -0.04 (-0.13, 0.07) \| 0.85 \| \| Mixed TPE (25%) and HE (75%) \| 0.61 (0.52, 0.72) \| -0.03 (-0.12, 0.08) \| 0.88 \| \| Mixed TPE (50%) and HE (50%) \| 0.62 (0.53, 0.72) \| -0.02 (-0.11, 0.08) \| 0.90 \| \| Mixed TPE (75%) and HE (25%) \| 0.63 (0.54, 0.73) \| -0.01 (-0.10, 0.09) \| 0.91 \| \| Pure TPE (100%) \| 0.64 (0.55, 0.73) \| -0.00 (-0.09, 0.10) \| 0.92 \| \| 1:1 allocation,  50% switching rate for control arm \| Pure HE (100%) \| 0.60 (0.51, 0.71) \| -0.04 (-0.13, 0.07) \| 0.85 \| \| Mixed TPE (25%) and HE (75%) \| 0.61 (0.53, 0.72) \| -0.03 (-0.12, 0.08) \| 0.88 \| \| Mixed TPE (50%) and HE (50%) \| 0.62 (0.54, 0.72) \| -0.02 (-0.11, 0.09) \| 0.90 \| \| Mixed TPE (75%) and HE (25%) \| 0.63 (0.55, 0.73) \| -0.01 (-0.09, 0.09) \| 0.91 \| \| Pure TPE (100%) \| 0.64 (0.56, 0.73) \| 0.00 (-0.09, 0.10) \| 0.92 \| \| **^a^This table shows estimated treatment effects under an assumed hazard ratio (HR) of 0.60 for the transition hazards of the illness-death model and bias and coverage in comparison to the true treatment policy estimand. Monte Carlo standard errors for all measures are very close to zero  Acronyms: CI: Confidence intervals; HE - Hypothetical estimator; HR - Hazard ratio; TPE - Treatment policy estimator.** \| \| \| \| \| |
| --- | --- | --- | --- | --- | --- | --- | --- | --- | --- | --- | --- | --- | --- | --- | --- | --- | --- | --- | --- | --- | --- | --- | --- | --- | --- | --- | --- | --- | --- | --- | --- | --- | --- | --- | --- | --- | --- | --- | --- | --- | --- | --- | --- | --- | --- | --- | --- | --- | --- | --- | --- | --- | --- | --- | --- | --- | --- | --- | --- | --- | --- | --- | --- | --- | --- | --- | --- | --- | --- | --- | --- | --- | --- | --- | --- | --- | --- | --- | --- | --- | --- | --- | --- | --- | --- | --- | --- | --- | --- | --- | --- | --- | --- | --- | --- | --- | --- | --- | --- | --- | --- | --- | --- | --- |

| Table 14: Averages of pooled treatment effect estimates and comparison against treatment policy estimand under an assumed HR of 0.80 for the transition hazards of the illness-death model in the simulation with random-effects data-generating mechanism, random-effects meta-analysis, and censoring switchers   \| **Scenarios** \| **Estimators** \| **Estimated treatment effects:  HR (averaged 95% CI)** \| **Bias  (2.5%, 97.5% difference percentiles)** \| **Coverage** \| \| --- \| --- \| --- \| --- \| --- \| \| **75% switching rate for control arm** \|  \|  \| **Comparison against treatment policy estimand (True HR = 0.84)** \| \| \| 2:1 allocation,  75% switching rate for control arm^a^ \| Pure HE (100%) \| 0.81 (0.66, 0.99) \| -0.03 (-0.18, 0.15) \| 0.91 \| \| Mixed TPE (25%) and HE (75%) \| 0.82 (0.69, 0.98) \| -0.02 (-0.16, 0.14) \| 0.91 \| \| Mixed TPE (50%) and HE (50%) \| 0.83 (0.71, 0.97) \| -0.01 (-0.14, 0.13) \| 0.92 \| \| Mixed TPE (75%) and HE (25%) \| 0.83 (0.72, 0.96) \| -0.01 (-0.12, 0.12) \| 0.92 \| \| Pure TPE (100%) \| 0.84 (0.73, 0.96) \| -0.00 (-0.11, 0.11) \| 0.92 \| \| 1:1 allocation,  75% switching rate for control arm \| Pure HE (100%) \| 0.81 (0.67, 0.98) \| -0.03 (-0.17, 0.14) \| 0.91 \| \| Mixed TPE (25%) and HE (75%) \| 0.82 (0.69, 0.97) \| -0.02 (-0.15, 0.13) \| 0.91 \| \| Mixed TPE (50%) and HE (50%) \| 0.83 (0.71, 0.96) \| -0.01 (-0.13, 0.12) \| 0.92 \| \| Mixed TPE (75%) and HE (25%) \| 0.83 (0.73, 0.96) \| -0.01 (-0.12, 0.12) \| 0.91 \| \| Pure TPE (100%) \| 0.84 (0.74, 0.95) \| -0.00 (-0.11, 0.12) \| 0.92 \| \| **50% switching rate for control arm** \|  \|  \| **Comparison against treatment policy estimand (True HR = 0.83)** \| \| \| 2:1 allocation,  50% switching rate for control arm \| Pure HE (100%) \| 0.80 (0.68, 0.95) \| -0.02 (-0.16, 0.13) \| 0.90 \| \| Mixed TPE (25%) and HE (75%) \| 0.81 (0.69, 0.95) \| -0.02 (-0.14, 0.12) \| 0.91 \| \| Mixed TPE (50%) and HE (50%) \| 0.81 (0.70, 0.95) \| -0.01 (-0.13, 0.12) \| 0.91 \| \| Mixed TPE (75%) and HE (25%) \| 0.82 (0.71, 0.95) \| -0.01 (-0.12, 0.12) \| 0.92 \| \| Pure TPE (100%) \| 0.82 (0.72, 0.95) \| -0.00 (-0.12, 0.12) \| 0.92 \| \| 1:1 allocation,  50% switching rate for control arm \| Pure HE (100%) \| 0.80 (0.68, 0.95) \| -0.02 (-0.15, 0.12) \| 0.90 \| \| Mixed TPE (25%) and HE (75%) \| 0.81 (0.69, 0.95) \| -0.02 (-0.14, 0.12) \| 0.91 \| \| Mixed TPE (50%) and HE (50%) \| 0.81 (0.70, 0.95) \| -0.01 (-0.13, 0.12) \| 0.92 \| \| Mixed TPE (75%) and HE (25%) \| 0.82 (0.71, 0.94) \| -0.01 (-0.12, 0.12) \| 0.91 \| \| Pure TPE (100%) \| 0.82 (0.72, 0.94) \| -0.00 (-0.11, 0.12) \| 0.91 \| \| **^a^This table shows estimated treatment effects under an assumed hazard ratio (HR) of 0.80 for the transition hazards of the illness-death model and bias and coverage in comparison to the true treatment policy estimand. Monte Carlo standard errors for all measures are very close to zero  Acronyms: CI: Confidence intervals; HE - Hypothetical estimator; HR - Hazard ratio; TPE - Treatment policy estimator.** \| \| \| \| \| |
| --- | --- | --- | --- | --- | --- | --- | --- | --- | --- | --- | --- | --- | --- | --- | --- | --- | --- | --- | --- | --- | --- | --- | --- | --- | --- | --- | --- | --- | --- | --- | --- | --- | --- | --- | --- | --- | --- | --- | --- | --- | --- | --- | --- | --- | --- | --- | --- | --- | --- | --- | --- | --- | --- | --- | --- | --- | --- | --- | --- | --- | --- | --- | --- | --- | --- | --- | --- | --- | --- | --- | --- | --- | --- | --- | --- | --- | --- | --- | --- | --- | --- | --- | --- | --- | --- | --- | --- | --- | --- | --- | --- | --- | --- | --- | --- | --- | --- | --- | --- | --- | --- | --- | --- | --- |

| Table 15: Averages of pooled treatment effect estimates and comparison against treatment policy estimand under an assumed HR of 1.00 for the transition hazards of the illness-death model in the simulation with random-effects data-generating mechanism, random-effects meta-analysis, and censoring switchers   \| **Scenarios** \| **Estimators** \| **Estimated treatment effects:  HR (averaged 95% CI)** \| **Bias  (2.5%, 97.5% difference percentiles)** \| **Coverage** \| \| --- \| --- \| --- \| --- \| --- \| \| **75% switching rate for control arm** \|  \|  \| **Comparison against treatment policy estimand (True HR = 1.00)** \| \| \| 2:1 allocation,  75% switching rate for control arm^a^ \| Pure HE (100%) \| 1.01 (0.82, 1.24) \| 0.01 (-0.18, 0.24) \| 0.94 \| \| Mixed TPE (25%) and HE (75%) \| 1.00 (0.84, 1.20) \| 0.00 (-0.16, 0.20) \| 0.93 \| \| Mixed TPE (50%) and HE (50%) \| 1.00 (0.86, 1.17) \| 0.00 (-0.15, 0.17) \| 0.93 \| \| Mixed TPE (75%) and HE (25%) \| 1.00 (0.86, 1.15) \| -0.00 (-0.14, 0.15) \| 0.93 \| \| Pure TPE (100%) \| 1.00 (0.87, 1.14) \| -0.00 (-0.13, 0.13) \| 0.93 \| \| 1:1 allocation,  75% switching rate for control arm \| Pure HE (100%) \| 1.02 (0.84, 1.24) \| 0.02 (-0.16, 0.23) \| 0.94 \| \| Mixed TPE (25%) and HE (75%) \| 1.01 (0.85, 1.20) \| 0.01 (-0.15, 0.19) \| 0.93 \| \| Mixed TPE (50%) and HE (50%) \| 1.00 (0.86, 1.17) \| 0.00 (-0.14, 0.17) \| 0.92 \| \| Mixed TPE (75%) and HE (25%) \| 1.00 (0.87, 1.15) \| 0.00 (-0.13, 0.15) \| 0.92 \| \| Pure TPE (100%) \| 1.00 (0.88, 1.14) \| -0.00 (-0.12, 0.13) \| 0.92 \| \| **50% switching rate for control arm** \|  \|  \| **Comparison against treatment policy estimand (True HR = 1.00)** \| \| \| 2:1 allocation,  50% switching rate for control arm \| Pure HE (100%) \| 1.00 (0.84, 1.19) \| 0.00 (-0.16, 0.19) \| 0.93 \| \| Mixed TPE (25%) and HE (75%) \| 1.00 (0.85, 1.18) \| -0.00 (-0.15, 0.18) \| 0.92 \| \| Mixed TPE (50%) and HE (50%) \| 1.00 (0.86, 1.17) \| -0.00 (-0.15, 0.16) \| 0.92 \| \| Mixed TPE (75%) and HE (25%) \| 1.00 (0.86, 1.15) \| -0.00 (-0.14, 0.15) \| 0.92 \| \| Pure TPE (100%) \| 1.00 (0.87, 1.15) \| -0.00 (-0.13, 0.15) \| 0.92 \| \| 1:1 allocation,  50% switching rate for control arm \| Pure HE (100%) \| 1.00 (0.85, 1.18) \| 0.00 (-0.16, 0.18) \| 0.92 \| \| Mixed TPE (25%) and HE (75%) \| 1.00 (0.86, 1.17) \| -0.00 (-0.15, 0.17) \| 0.92 \| \| Mixed TPE (50%) and HE (50%) \| 1.00 (0.86, 1.16) \| -0.00 (-0.14, 0.16) \| 0.92 \| \| Mixed TPE (75%) and HE (25%) \| 1.00 (0.87, 1.15) \| -0.00 (-0.14, 0.15) \| 0.92 \| \| Pure TPE (100%) \| 1.00 (0.87, 1.14) \| -0.00 (-0.13, 0.14) \| 0.92 \| \| **^a^This table shows estimated treatment effects under an assumed hazard ratio (HR) of 1.00 for the transition hazards of the illness-death model and bias and coverage in comparison to the true treatment policy estimand. Monte Carlo standard errors for all measures are very close to zero  Acronyms: CI: Confidence intervals; HE - Hypothetical estimator; HR - Hazard ratio; TPE - Treatment policy estimator.** \| \| \| \| \| |
| --- | --- | --- | --- | --- | --- | --- | --- | --- | --- | --- | --- | --- | --- | --- | --- | --- | --- | --- | --- | --- | --- | --- | --- | --- | --- | --- | --- | --- | --- | --- | --- | --- | --- | --- | --- | --- | --- | --- | --- | --- | --- | --- | --- | --- | --- | --- | --- | --- | --- | --- | --- | --- | --- | --- | --- | --- | --- | --- | --- | --- | --- | --- | --- | --- | --- | --- | --- | --- | --- | --- | --- | --- | --- | --- | --- | --- | --- | --- | --- | --- | --- | --- | --- | --- | --- | --- | --- | --- | --- | --- | --- | --- | --- | --- | --- | --- | --- | --- | --- | --- | --- | --- | --- | --- |
